# Detection and Analysis of the Bacterium, *Xylella fastidiosa*, in Glassy-Winged Sharpshooter, *Homalodisca vitripennis*, Populations in Texas

**DOI:** 10.1673/031.010.14128

**Published:** 2010-10-04

**Authors:** Daymon Hail, Forrest Mitchell, Isabelle Lauzière, Patrick Marshall, Jeff Brady, Blake Bextine

**Affiliations:** ^1^ University of Texas at Tyler, 3900 University Blvd, Tyler, TX 75799; ^2^ Texas AgriLife Research, 1229 N US Hwy 281, Stephenville, TX 76401; ^3^ Texas Pierce's Disease Research & Education Program, Texas AgriLife Research, 259 Business Court Fredericksburg, TX 78624

**Keywords:** insect vector, plant pathogen, Pierce's disease

## Abstract

The glassy-winged sharpshooter, *Homalodisca vitripeninis* Germar (Hemiptera: Cicadellidae), is a xylophagous insect that is an endemic pest of several economically important plants in Texas. *H. vitripennis* is the main vector of *Xylella fastidiosa* Wells (Xanthomonadales: Xanthomonadaceae), the bacterium that causes Pierce's disease of grapevine and can travel long distances putting much of Texas grape production at risk. Understanding the movement of *H. vitripennis* populations capable of transmitting *X. fastidiosa* into Pierce's-disease-free areas is critical for developing a management program for Pierce's disease. To that end, the USDA-APHIS has developed a program to sample vineyards across Texas to monitor populations of *H. vitripennis.* From this sampling, *H vitripennis* collected during 2005 and 2006 over the months of May, June, and July from eight vineyards in different regions of Texas were recovered from yellow sticky traps and tested for the presence of *X. fastidiosa.* The foregut contents were vacuum extracted and analyzed using RT-PCR to determine the percentage of *H. vitripennis* within each population that harbor *X. fastidiosa* and have the potential to transmit this pathogen. *H. vitripennis* from vineyards known to have Pierce's disease routinely tested positive for the presence of *X. fastidiosa.* While almost all *H. vitripennis* collected from vineyards with no history of Pierce's disease tested negative for the presence of the pathogen, three individual insects tested positive. Furthermore, all three insects were determined, by DNA sequencing, to be carrying a strain of *X. fastidiosa* homologous to known Pierce's disease strains, signifying them as a risk factor for new *X. fastidiosa* infections.

## Introduction

The glassy-winged sharpshooter, *Homalodisca vitripeninis* Germar (Hemiptera: Cicadellidae), is an insect pest that is present in most of the southern region of the USA and is an endemic pest throughout most regions of Texas (Young 1958; Turner and Pollard 1959). Without naturally occurring forms of biological control, *H. vitripennis* have established populations in southern California and have negatively affected the wine grape industry (Founier et al. 2003). With the ability to travel long distances, *H. vitripennis* can spread quickly once established and have recently been found in French Polynesia, Tahiti, and Hawaii (Hoddle et al. 2003).

*H. vitripennis* have the ability to ingest in excess of 100 times their weight in xylem fluid in a single day (Purcell 1999). They have been reported to feed on host plants from at least 35 families, including both woody and herbaceous types (Hoddle et al. 2003). *H. vitripennis* feeding can impact plant health directly by depriving the plant of nutrients and damaging the xylem sufficiently to preclude vascular flow. Indirectly, plant damage is done by the transmission of the xylem-limited bacterium, *Xylella fastidiosa* Wells (Xanthomonadales: Xanthomonadaceae).

*X. fastidiosa* infection in grapevines may result in Pierce's disease, which has caused major losses in both wine and table grape production in the USA (Davis et al. 1978). In the grapevine (*Vitis* sp.), Pierce's disease symptoms include marginal leaf scorch, chlorosis, necrosis, stunted growth, leaf loss, and dieback, all of which result from occlusion of the xylem tissue by polymeric matrix enclosed bacterial aggregates attached to the inner xylem wall (Hopkins 1989). *X.* *fastidiosa* can cause systemic failure of a grapevine within one to five years of initial infection, and previous studies have shown that as few as 100 cells can initiate an infection (Hill and Purcell 1995). As there is currently no cure for Pierce's disease (Pooler et al. 1997), grapevines showing characteristic symptoms must be uprooted and replanted, usually resulting in a two or three year loss of individual plant productivity.

Many economically important plants includeing citrus, almond and oleander are affected by separate strains of *X. fastidiosa,* resulting in a multitude of plant diseases such as citrus variegated chlorosis (Chang et al. 1993; Pooler and Hartung 1995), almond leaf scorch (Mircetich et al. 1976), and oleander leaf scorch (Purcell 1999). Many strains of *X. fastidiosa* are host specific and in transmission studies, the strain that causes disease symptoms in oleander will not cause disease symptoms in grape or almond. Additionally, the grape and almond strains were unable to cause disease symptoms in oleander.

Greenhouse studies suggest that between 10% and 20% of *H. vitripennis* are able to transmit *X. fastidiosa* (Almeida and Purcell 2006), but there is little data on naturally occurring infectivity (Daane et al. 2007). Many methods have been developed to detect *X. fastidiosa* in natural and experimental environments including transmission (Purcell and Finlay 1980), insect head culture (Almeida and Purcell 2003), plant tissue culture (Hill and Purcell 1995), chloroform/phenol extraction (Frohme et al. 2000), and PCR-based vacuum extraction (Bextine et al. 2005). Culture-based detection methods are difficult and time consuming given the fastidious nature of the bacterium and are inherently less sensitive than PCR based techniques. RT-PCR can be used to detect as few as five *X. fastidiosa* cells in an insect head (Bextine et al. 2005) and is a viable approach even when dealing with dead insects, making it a highly valuable procedure when compared to other forms of detection.

In *X. fastidiosa, gyrase B* is conserved in all strains and is diverse enough to also be used as a molecular marker for both detection and strain differentiation (Bextine et al. 2005). In this study, eight vineyards from different regions of Texas were surveyed for the presence of *H. vitripennis*, and potential vectors were tested for the presence and strain of *X. fastidiosa.*

**Figure 1.**
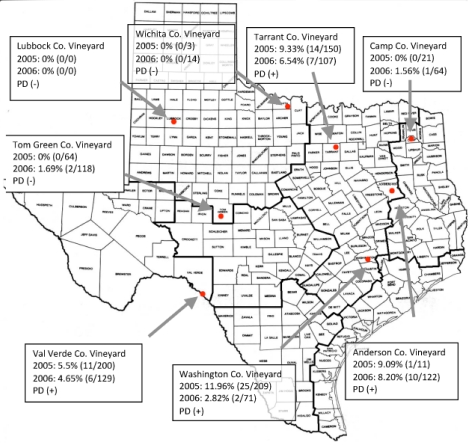
Regional map of Texas showing collection locations in counties (Co.) sampled. Percentages shown reflect *Xylella fastidiosa* infectivity for 2005 and 2006. Also shown are independent results of Pierce's disease (PD) known for each vineyard. High quality figures are available online.

## Materials and Methods

### Sample preparation

Eight vineyards in different regions of Texas, Washington County, Anderson County, Camp County, Tarrant County, Wichita County, Lubbock County, Tom Green County and Val Verde County (Figure 1), were sampled for the presence of *X. fastidiosa* vector species. In an effort to prevent negative business consequences as a result of this report and so that the identities of the vineyards in question would not affect the outcome of this study, the names and exact locations of the vineyards were kept anonymous. Monitoring of insect populations took place using standard doublesided traps (Seabright Laboratories, www.seabrightlabs.com), each 23 × 14 cm in size, bright yellow in color (Pantone® Matching System (PMS) 102) and coated with Stikem Special® glue (www.seabrightlabs.com). Traps were tightly stapled to a 1.8 m bamboo stake driven into the ground a little lower than grapevine canopy. Between 6 and 13 traps were placed in each vineyard (Lauziere et al. 2008).

Upon retrieval from the vineyard, the traps were placed into Ziploc bags and stored at 4° C. The traps were then removed from the bags, and *H. vitripennis* were removed by applying the solvent orange oil (Citrus King, www.citrusdepot.net) around the insect to dissolve the adhesive and remove the insect from the trap. Each insect was then washed in 95% ethanol and then in deionized water to remove any residual orange oil. Insect heads were removed (Bextine et al. 2004), and a silica-based DNA extraction was performed to test for the presence of *X. fastidiosa.*

### DNA extraction

Each head was placed into a well of a 96-well plate (VWR International, www.vwrsp.com) and submerged in 100 µl of PBS buffer. Vacuum pressure was applied to the plate four times for two minutes each (Bextine 2004). With *X. fastidiosa* cells dislodged during vacuum extraction, the heads were discarded and the vacuum solution was retained. To each well, 100 µl of TE buffer (5.25 *M* guanadine thiocyanate, 50 mM Tris-Cl (pH 6.8), 20 mM EDTA (pH 8.0) and 1.3% w/v Triton X-100) was added to lyse bacterial cells. The contents were mixed by pipetting, and the mixture was centrifuged for 5 min at 5000 rpm to separate DNA from the cellular debris. The contents of each well were then transferred into the corresponding wells of 0.2 ml eight-well strips, and 53 µl of silica slurry (molecular grade H_2_O and silicon dioxide, S5631 Sigma-Aldrich, www.sigmaaldrich.com) were added and mixed by pipetting. The eight-well strips were then returned to their corresponding rows in a 96-well plate. The plate was incubated at room temperature for 5 min and centrifuged at 2000 rpm for 5 min. The supernatant was then discarded, and the DNA pellet was retained. DNA pellets were washed four times by resuspending the silica in 200 µl wash buffer (40% vol/vol 100 mM Tris-Cl (pH 7.5), 20 mM EDTA (pH 8.0), 0.4 *M* NaCl and 60% vol/vol 100% EtOH) and centrifuging for 5 min at 2000 rpm. After the wash buffer was removed, the resulting pellets were dried in an incubator at 60° C for 10 min. The silica was resuspended in 100 µl of TE Buffer and incubated again for 5 min at 60° C, followed by a final centrifuge for 5 min at 5000 rpm. Carefully avoiding the silica, 70 µl of the resulting DNA elution were removed. From each sample, 2 µl were removed and analyzed at 230 nm in a NanoDrop 1000 spectrophotometer (Thermo Scientific, www.thermoscientific.com) to quantify the extracted DNA.

### *Xylella fastidiosa* detection

A SYBR-green based Real Time-PCR (RT-PCR) was performed on the subsequent elutions using the *X. fastidiosa* specific primers (Bextine and Child 2007) (INF2 5′-GTTTGATTGATGAACGTGGTGAG and INR1 5′-CATTGTTTCTTGGTAGGCATCA G) designed for the Gyrase B gene. A master mix was made using 10 µl of IQ Supermix (BioRad, www.bio-rad.com), 0.8 µl of both primers (at a concentration of 10 µM), 5.4 µl of autoclaved molecular grade water, 1 µl of 10 µM Sybr Green (Invitrogen, www.invitrogen.com) and 2 µl DNA template per reaction. The run conditions for the PCR were 95° C for 3 min; then 40 cycles of 95° C for 20 sec, 55° C for 30 sec, and 72° C for 60 sec; followed by DNA melting temperature curve analysis which ramped from 77–90° C by 0.5° C each step. As a positive control, DNA was extracted from PD3 (Davis et al. 1978) plated *X. fastidiosa* by the methods described above.

### *Xylella fastidiosa* strain differentiation

Another SYBR-Green based RT-PCR was performed on the *H. vitripennis* testing positive for *X. fastidiosa* using GyrBLONG primers (GryBLONG F3 5′-CATCCAAGTTGCTTCTGCAC and GyrBLONG R2 5′-GTTATAAGCAGCCGGACCTG). A master mix was made using 10 µl of IQ Supermix (BioRad), 0.8 µl of both primers (at a concentration of 10 µM), 5.4 µl of autoclaved molecular grade water, 1 µl of 10 µM Sybr Green (Invitrogen) and 2 µl DNA template per reaction. The run conditions for the PCR were 95° C for 3 min; then 40 cycles of 95° C for 30 sec, 53° C for 60 sec, and 72° C for 120 sec; followed by DNA melting temperature curve analysis which ramped from 70–99° C by 0.1° C each step. As a positive control, DNA was extracted from PD3 (Davis et al. 1978) plated *X. fastidiosa* by the methods described above.

The positive PCR products were purified using the QIAquick PCR purification kit (Qiagen, www.qiagen.com) according to the manufacturer's protocol. A forward and a reverse DNA-sequencing PCR was performed in triplicate in a 10-µl reaction containing 4 µl of DTCS Quick Start Master Mix (Beckman Coulter, www.beckmancoulter.com), 2 µl of either the forward or reverse primer (10 µM), 2 µl of autoclaved nanopure water, and 2 µl of a DNA template. The sequencing PCR (30 cycles) was conducted under the following conditions: 95° C for 20 s, 50^0^C for 20 s, and 60°C for 4 min, with the product then held at 4° C until removal from the machine. The DNA product was purified using standard ethanol precipitation and resuspended in 40 µl of sample-loading solution (Beckman Coulter).

The resuspended samples were transferred to the appropriate Beckman Coulter 96-well microplates, centrifuged at 300 rpm at 2°C for 30 s, and then overlaid with one drop of mineral oil. Samples then were sequenced in a CEQ 8000 Genetic Analysis System (Beckman Coulter) using the manufacturer's protocol. The resulting DNA sequences were translated into amino acid sequences using ExPASy's (www.expasy.ch) translate tool. The stop codons were then edited out and a consensus sequence was created using BioEdit's sequence editing tools (Ibis Biosciences, www.ibisbiosciences.com). The trimmed and edited DNA sequences were matched to known sequences using NCBI's megablast algorithm (Basic Local Alignment Search Tool — BLAST; www.ncbi.nih.gov). Results were compared using a χ^2^ analysis and a Students *t*-test.

## Results

As expected, some vineyards were more heavily infested with *H. vitripennis* than others. The vineyards in Val Verde and Washington counties had as many as 200 or more *H. vitripennis* recovered from their yellow-sticky cards, whereas the vineyards in Tarrant and Tom Green counties had around 100. More northern vineyards (Wichita and Camp) had as few as three and as many as a few dozen individuals, while the vineyard in Lubbock county, the only vineyard sampled on the High Plains, had no *H. vitripennis* at all (Table 1). While this trend may seem concordant with the presence or absence of Pierce's disease in a vineyard, there were exceptions and irregular sampling at various sites. The Anderson county vineyard was sampled only once in 2005, which prevented statistical analysis of this site.

**Table 1.**
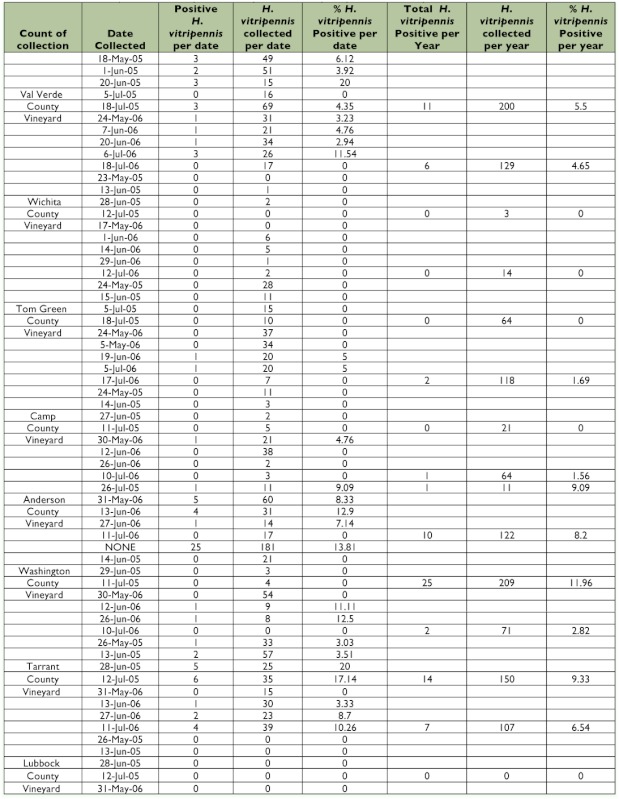
*Homalodisca vitripennis* tested for presence of *Xylella fastidiosa* by vineyard & collection period.

**continued:**
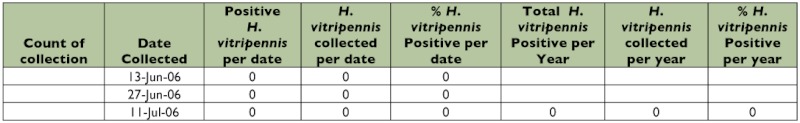

The range of concentrations of DNA in all samples testing positive for *X. fastidiosa* was from 3.2 to 36.3 ng/µl. Of those *H. vitripennis* collected, many tested negative for the presence of *X. fastidiosa*. Taking all vineyards into account, the percentage positive was 6.16 (79/1283); this is a lower percentage than the estimation given by Almeida and Purcell (2003). The highest percentage positive occurred in the same vineyard as the highest number of individuals positive (Washington county vineyard, 11.96%, 25/209). Other vineyards such as those in the Anderson and Tarrant counties had similar percentage positives (9.09 and 9.33, respectively), but lower *H. vitripennis* counts. The northern-most vineyards (Wichita, Lubbock, and Camp) tended to have not only the lowest numbers of individuals, but also the lowest percentages positive for both years sampled (0/17, 0/0, and 1/85 respectively). No vineyard was significantly different from one year to another when comparing 2005 data to 2006 data (Val Verde: p = 0.455; Wichita: p = 0.499; Tom Green: p = 0.476; Camp: p = 0.485; Anderson: p = 0.448; Washington: p = 0.395; Tarrant: p = 0.397; Lubbock could not be calculated due to zero values overall). The number of *H. vitripennis* testing positive or negative for *X. fastidiosa* was significantly different across vineyards (χ^2^: 26.602; df = 6; p < 0.001), and vineyards with a history of Pierce's disease had more *H. vitripennis* testing positive for *X. fastidiosa* than did those vineyards without a history of Pierce's disease (7.61% compared to 1.06%, respectively; *t* = 0.02). For almost all samples that tested positive for the presence of *X. fastidiosa, gryB* sequences were identical to other known Pierce's disease strains.

Samples that tested positive for the presence of *X. fastidiosa* from vineyards that had no history of Pierce's disease were reanalyzed to determine strain through DNA sequencing. Results from the melt curve analysis were difficult to interpret because of primer dimer, but were consistent with sequencing results. Sequence data from the *gyrB* and *mopB* genes were analyzed to determine the strain of *X. fastidiosa* that was detected within the vector insects (Morano et al. 2008). Three *H. vitripennis* that were collected from vineyards that were considered Pierce's disease-free (Wichita, Tom Green, and Camp) were determined to contain the Pierce's disease strains through *gyrB* sequence analysis (Table 2).

## Discussion

Screening of samples was conducted using the INF2 and INR1 primer set. These primers were originally designed to differentiate between strains of *X. fastidiosa* through melt curve analysis; however, the results were difficult to interpret due to the influence of primer dimer that caused some minimal overlapping melt temperatures between strains. Using GyrBLONG primers (another primer set that is being developed for *X. fastidiosa* strain identification), it was determined that while the majority of samples tested negative for *X. fastidiosa* (∼93%), some of the *H. vitripennis* testing positive for *X. fastidiosa* contained an ornamental strain (*X. fastidiosa* multiplex) (Schaad et al. 2004). Strains of *X. fastidiosa* are specific with respect to their role in pathogenicity; thus, colonization of grapevines by an ornamental strain (*X. fastidiosa* multiplex or *X. fastidiosa* sandyi) (Schaad et al. 2004) will not result in the overt symptomology typical among Pierce's disease infections (Almeida and Purcell 2003).

Although the results reported here are reasonably consistent with greenhouse infectivity estimates published by Almeida and Purcell (2003), there is always the possibility of false negatives or false positives due to inconclusive melt temperature analysis or other factors. Some potential false negatives could be the result of trap exposure to the elements. The two-week period in which the traps were exposed to cycles of mid-day heating and nighttime cooling could have led to degradation of *X. fastidiosa* DNA. However, in previous studies, DNA was recoverable and detection by PCR was competent from insects on traps that were exposed to the elements in southern California as long as 10 days, the longest period tested (Bextine et al. 2005). False negatives likely did not compromise the integrity of this study.

**Table 2.**
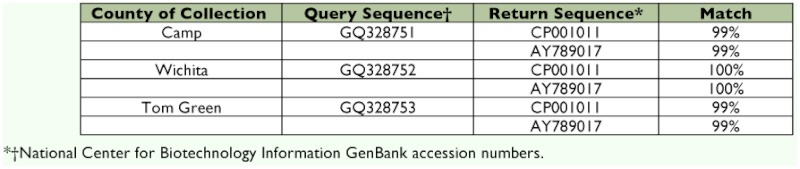
Sequence data obtained from the Gyrase B gene of *Xylella fastidiosa* isolated from *Homalodisca vitripeninis* collected in three vineyards previously considered Pierce's disease free (Camp County, Wichita County and Tom Green County).

Many *H. vitripennis* collected within vineyards with a documented history (in Anderson, Tarrant, and Washington counties) tested positive for the Pierce's disease strain of *X. fastidiosa.* This was not surprising, given the presence of this bacterial strain in the immediate plant community. However, the detection of the non-Pierce's disease strain in insects collected from vineyards with a known history of Pierce's disease was not expected but supports findings by Lauzière and others of migration between vineyard and nonvineyard wild populations. This is an interesting finding, given the availability of the Pierce's disease strain of *X. fastidiosa.* Costa et al. (2003) found that *H. vitripennis* that came in contact with an ornamental strain of *X. fastidiosa* prior to coming in contact with the Pierce's disease strain of the bacterium are less likely to acquire and transmit the grapevine pathogen. Therefore, *H. vitripennis* carrying an ornamental strain of the bacterium may present limited risk as a potential vector when it enters a vineyard. However, *H. vitripennis* that tested positive for a non-Pierce's disease strain of the bacterium represented a very small percentage of the insects collected that tested positive for the presence of *X. fastidiosa.*

Perhaps the most imminent threat posed by *H. vitripennis* that was uncovered in this study was the identification of insects carrying the Pierce's disease strain in vineyards with no history of disease. At the time of this study, Lubbock county, Camp county, and Tom Green county vineyards had no documented history of Pierce's disease infection; however, *X. fastidiosa-*positive *H. vitripennis* were identified in the latter two. This result could be due to migratory or wind-driven individuals moving from vineyards in areas with a history of Pierce's disease or from wild hosts infiltrating the area. When these samples were genotyped using a RT-PCR analysis and direct sequencing of pathogen DNA, the samples were determined to contain the Pierce's disease strain of the bacterium. While these individuals may seem to be only a small proportion the *H. vitripennis* found in these vineyards, a single infective insect could be responsible for the primary spread of the bacterium into a new area.

In the time since the completion of this study, the Camp County vineyard and Tom Green county vineyard have become Pierce's disease hot spots. This result was expected since the identification of *X. fastidiosa* infected *H. vitripennis* reported in this study. Lubbock county vineyard has also since become a Pierce's disease hot spot; although, at this time it does not appear to have been caused by a traditional epidemiological situation. Further analysis of these insects is needed to determine their point of origin, possibly leading to effective methods for limiting the threat they pose. Continued year-round sampling in all vineyards in years to come will allow researchers to better isolate peaks (or shifts of peaks) of infectivity and over-wintering sites for *H. vitripennis.*

## Abbreviation

RT-PCR: real time PCR
